# The extent and barriers in providing pharmaceutical care services by community pharmacists in Malaysia: a cross-sectional study

**DOI:** 10.1186/s12913-021-06820-7

**Published:** 2021-08-16

**Authors:** Pengyeow Loh, Siew Siang Chua, Mahmathi Karuppannan

**Affiliations:** 1 School of Pharmacy, Faculty of Health & Medical Sciences, Taylor’s University, Subang Jaya, Selangor Malaysia; 2 Department of Pharmacy Practice, Faculty of Pharmacy, Universiti Teknologi MARA Cawangan Selangor, Puncak Alam Campus, Bandar Puncak Alam, Selangor Malaysia

**Keywords:** Community pharmacists, Pharmaceutical care, Pharmacy services, Barriers

## Abstract

**Background:**

Since the introduction of pharmaceutical care concept by Hepler and Strand in 1990, community pharmacists worldwide have been realigning their roles from being product-focused to patient-orientated to improve patient's quality of life. The objectives of this study were to determine the type of services, with emphasis on the extent of pharmaceutical care services provided by community pharmacists and the barriers in providing such services in Malaysia.

**Methods:**

A cross-sectional observational study was conducted using an online questionnaire. Community pharmacists in Malaysia were invited to participate in the study via emails. The questionnaire was structured based on the Malaysian Community Pharmacy Benchmarking Guidelines, the five practice principles of pharmaceutical care by the American Pharmacists Association and other studies. The online questionnaire was opened for 6 months, from April to September 2018. A reminder to participate was sent via email to the community pharmacists every fortnight.

**Results:**

A total of 420 community pharmacists responded to the online questionnaire. Besides essential services such as treatment for minor illness, medicine dispensing and counselling, most of the respondents were providing health screening and monitoring (99.5%), selection and recommendation of health supplements (90.5%), patient medication review (68.8%), weight management (52.4%) and counselling on smoking cessation (51.0%). More than half (53.3%) of the respondents reported that they were providing pharmaceutical care services to patients with chronic diseases. Based on the practice principles of pharmaceutical care, the respondents were involved in patients' data collection (23.3%), medical information evaluation (18.6%), formulating a drug therapy plan (9.3%), implementing a drug therapy plan (4.5%), and monitoring and modifying the plan (18.3%). Lack of separation between prescribing and dispensing was perceived as the main barrier to the implementation of pharmaceutical care services by a majority of the respondents (84.0%).

**Conclusions:**

The present study found that pharmaceutical care services provided by community pharmacists in Malaysia were inadequate compared to international practice principles. Areas that need improvement included collaboration with patients' other health care providers; more proactive management of patient's medicine regimen; having proper patient monitoring and follow-up mechanisms, and documentation.

**Supplementary Information:**

The online version contains supplementary material available at 10.1186/s12913-021-06820-7.

## Background

Since the introduction of the pharmaceutical care philosophy by Hepler and Strand in 1990, community pharmacists worldwide have been urged by the respective governments and professional bodies, to shift their roles from being product-focused to patient-orientated, to achieve definite outcomes that improve a patient's quality of life [1]. The American Pharmacists Association (APhA) developed five principles on the practice of pharmaceutical care to ensure positive outcomes for patients. These five principles included: patients’ data collection; medical information evaluation; formulating a drug therapy plan; implementing the plan; monitoring and modifying the plan [2].

Studies on pharmaceutical care services provided by community pharmacists found that such services improved adherence to medications for chronic diseases such as hypertension, osteoporosis and hyperlipidaemia [3–5]. The optimisation of medication use had also led to a reduction in health care cost [4, 5]. Patients receiving pharmaceutical care services showed improved health indices such as significant decreases in glycated haemoglobin (HbA1c) level, systolic and diastolic blood pressure [6–9], better knowledge of the disease and improvement in certain components of health-related quality of life [8], as well as experienced a reduction in length of hospital stay and a lower rate of hospital readmission [10–12].

However, various studies had raised concern on the under-utilisation of community pharmacists in Malaysia in providing optimal health care to the general public and contributing to the health care system of the country [13–15]. Community pharmacists in Malaysia were mainly involved in supplying medicines and health supplements, providing advice and treatment for minor health problems, and conducting health screening tests [16], although some also provided health promotion services such as counselling on healthy lifestyles, smoking cessation and weight management [13, 17]. Thus far, there is a lack of recent studies on the types of pharmacy services provided by community pharmacists nationwide. Besides, studies specifically on pharmaceutical care services provided by community pharmacists in Malaysia, as well as possible barriers for community pharmacists to practise such services are still lacking. Sam et al. concluded that slightly more than half of the patients were satisfied with the pharmaceutical care services provided by a community pharmacist [18]. Another Malaysian study showed positive clinical outcomes of diabetes patients who went through a community pharmacist-based diabetes management programme [7]. However, both studies were limited to only one community pharmacy in Malaysia. Other local studies on pharmaceutical care focused more on counselling provided by community pharmacists [13, 19, 20]. Although medication counselling is an important element of pharmaceutical care and has been shown to have a positive impact on patient medicine use behaviour and adherence [21], this is only a part of the practice principles for pharmaceutical care by APhA [22].

At the time of the present study, there were more than 2,500 community pharmacies in Malaysia [23]. The health care resources in Malaysia are already stretching to the limit. As community pharmacies are easily accessible by the general public, they should be fully utilised in the development and implementation of the health care system in Malaysia. The question is: Are community pharmacists providing adequate pharmaceutical care services which can contribute to the health care system in Malaysia?

Therefore, the main objectives of the present study were to determine the types of services provided by community pharmacists in Malaysia, with emphasis on the extent of pharmaceutical care services and any barriers associated with the provision of such services, from the pharmacists' perspectives. The findings of this study will enable health care stakeholders in the country to better understand and plan a more comprehensive health care system in Malaysia. This should involve all health care providers including community pharmacists in the partnership or integration between public and private health sectors [24]. This study will also bring awareness to community pharmacists on areas of pharmacy practice which they need to prioritise or improve to provide better health care. In addition, the insights on perceived barriers by the community pharmacists in the implementation of pharmaceutical care will enable related professional bodies to continue engaging with policymakers to develop a better platform for the profession to contribute to the health care system of the country.

## Methods

This was a cross-sectional study which used an online, self-administered questionnaire via Survey Monkey®. The target population was community pharmacists who were working in private community pharmacies on a full-time basis in the country. Pharmacists who were working on part-time basis as locum pharmacists, or only in the administration or wholesale department of a community pharmacy, or a community pharmacy located inside a hospital premise, were excluded based on a screening question placed at the beginning of the questionnaire.

Using Raosoft® sample size calculator (http://www.raosoft.com /samplesize.html), with 95% confidence level, 5% margin of errors, a study population size of 2,278 and assuming that the prevalence of pharmaceutical care provision was 50%, the calculated sample size was 329.

A questionnaire was developed based on the Community Pharmacy Benchmarking Guideline [25] as well as the five practice principles of pharmaceutical care [2] and from other studies in the literature [13, 16, 17, 26]. The questionnaire used in this study was developed specifically for this study and provided in the Additional File 1.

The evaluation of pharmaceutical care services provided was based on the five practice principles of pharmaceutical care by the American Pharmacists Association (APhA) [2]: (1) Patients' data collection, (2) Medical information evaluation, (3) Formulating a drug therapy plan, (4) Implementing a drug therapy plan, (5) Monitoring and modifying the plan. These five practice principles were evaluated using 15 sub-questions to best match the definition of each principle by APhA [2].

Ten practising community pharmacists with 10 to 20 years of working experience were consulted on which sub-questions could be used to fulfil each of the pharmaceutical care practice principle by APhA [2]. The response and feedback were analysed and the research team concluded that the community pharmacists needed to carry out all the sub-questions under each of the practice principles to be considered as having fulfilled the practice principle concerned (Additional File 2).

Content validity and clarity of the questionnaire were reviewed and assessed by five practising community pharmacists who have a minimum of 10 years working experience. The questionnaire was modified based on their feedback and uploaded online using Survey Monkey®. A pilot study was conducted on 20 community pharmacists. These community pharmacists consisted of those in Northern (5), Central (6), Southern (3), East Coast (2) regions of Peninsular Malaysia, and from East Malaysia (4). These community pharmacists were requested not to participate in the main study. The results of the pilot study were excluded from the main study.

A list of email contacts of registered community pharmacists in Malaysia was compiled with the help of the Malaysian Pharmaceutical Society (MPS). Community pharmacists were contacted via email to participate in this study. The study was conducted using an online survey. When the invited pharmacists clicked the survey link included in the emails sent to them, they would be connected to the information sheet which contained information about the study, estimated time to complete the survey, participants’ rights to withdraw from the study at any time, including withdrawal of any information provided, and the assurance of their confidentiality. If an invited pharmacist agreed to participate in the study, he/she was required to click a checkbox for consent before he/she could proceed to do the questionnaire. The study had been reviewed and approved by the Human Ethics Committee of Taylor’s University Malaysia (reference number: HEC 2018/002).

The anonymity of the respondents was preserved in the study using the “anonymous responses collector” option from Survey Monkey® so as not to track and store the respondents' information. The “multiple responses” option in Survey Monkey® was turned off to allow only one response per browser or email address, thus minimising multiple responses from a single respondent. The online questionnaire was opened for 6 months, from April to September 2018. During these 6 months, a reminder to participate was sent every fortnight via email to only non-responders or partial responders using a feature in the SurveyMonkey.

### Data analysis

All data were entered and analysed using the Statistical Package for the Social Sciences (SPSS) version 21 (IBM Corp, Armonk, New York). Descriptive statistics were performed on all variables. For numeric data, the mean (standard deviation, SD) and median (interquartile range, IQR) were generated. Any possible associations between variables were first tested using univariate chi-square analysis and variables that produced p-value equal to or less than 0.25 were further tested using binary logistic regression under the generalised linear model function [27]. A p-value of less than 0.05 was considered as statistically significant. A small Chi-square value with *p* > 0.05 from Hosmer-Lemeshow Goodness of Fit test indicates that the model fit is acceptable [28].

Barriers in implementing pharmaceutical care were graded based on a 6-point Likert-like scale, from strongly disagree (1), disagree (2), slightly disagree (3), slightly agree (4), agree (5) to strongly agree (6). Mean score (SD) was calculated for each barrier statement [29]. These responses were then collapsed into three groups: ‘strongly disagree and disagree’, ‘slightly disagree and slightly agree’ and ‘agree and strongly agree’ to ease interpretation of results [29, 30].

## Results

From the list of 2,278 community pharmacists, 52 responded that they had stopped practising as community pharmacists while two invalid email addresses were returned, leaving a total of 2,224 community pharmacists. The online questionnaire survey was closed after 6 months with 420 completed questionnaires. This gives a response rate of 18.9% (420/2,224) but meets the minimum required number of 329 from the Raosoft® calculation. Characteristics of the respondents are as shown in Table 1. The proportion of respondents from the various states was similar to the distribution of community pharmacies in each state of Malaysia.

**Table 1:** Characteristics of respondents and practice premises (*N* = 420)

Characteristics	Number of respondents (%)
Sex	
Female	264 (62.9%)
Male	156 (37.1%)
Age group	
21-29	80 (19.0%)
30-39	150 (35.7%)
40-49	118 (28.1%)
50-59	57 (13.6%)
60 and above	15 (3.6%)
Ethnic group	
Malay	84 (20.0%)
Chinese	309 (73.6%)
Indian	16 (3.8%)
Others^a^	11 (2.6%)
Highest pharmacy related education level	
Basic pharmacy degree	399 (95.0%)
Master’s degree	21 (5.0%)
Ph.D in Pharmacy	0 (0.0%)
Country of basic degree	
Malaysia	244 (58.1%)
Overseas	176 (41.9%)
Years of practice in Malaysia	
Less than 5	124 (29.5%)
5 to 9	77 (18.3%)
10 to 14	53 (12.6%)
15 to 19	67 (16.0%)
20 to 24	62 (14.8%)
25 and above	37 (8.8%)
Type of Employment	
Self-employed / Shareholder	187 (44.5%)
Full time employee	233 (55.5%)
Premise location (state)	
Selangor	94 (22.4%)
Johor	55 (13.1%)
Kuala Lumpur	51 (12.1%)
Sarawak	45 (10.7%)
Penang	37 (8.8%)
Sabah	30 (7.1%)
Perak	27 (6.4%)
Negeri Sembilan	21 (5.0%)
Pahang	15 (3.6%)
Malacca	13 (3.1%)
Kedah	10 (2.4%)
Kelantan	7 (1.7%)
Terengganu	7 (1.7%)
Putrajaya	5 (1.2%)
Perlis	2 (0.5%)
Labuan	1 (0.2%)
Number of prescriptions/year	
0	21 (5.0%)
1-50	302 (72.0%)
51-100	45 (10.7%)
101-500	42 (10.0%)
501-1,000	3 (0.7%)
1,000-5,000	6 (1.4%)
5,001-10,000	1 (0.2%)

^a^Others includes Bidayuh, Gujarati, Kadazhan, Murut, mixed ethnic and undisclosed

### Types of pharmacy services provided

**Fig. 1.**
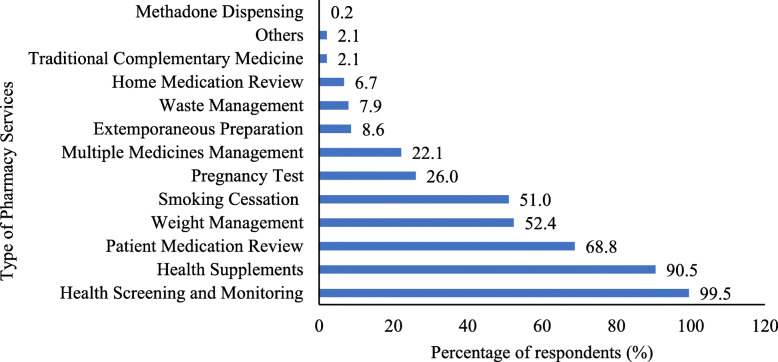
Types of pharmacy services provided regularly other than essential services (*N* = 420). Note: The percentage may exceed 100% as the respondents can provide more than one type of service. Others included pain and wound management, counselling on breastfeeding, diet and healthy lifestyle

Figure 1 shows the types of pharmacy services provided by the respondents regularly, besides essential services such as response to minor illnesses, medicine dispensing and medication counselling, as stipulated in the Community Pharmacy Benchmarking Guideline [25].

Almost all the respondents (99.5%) were providing at least one type of health screening and monitoring, with measurement of blood pressure (99.3%), blood glucose (97.9%), and cholesterol (74.5%) levels as the most common services.

Among the respondents who offered the selection and recommendation of health supplements for general health, only 15.8% (60) have been certified with Complementary Medicine Education (CMed). Whereas, of the respondents who were providing smoking cessation programme to assist their clients to quit smoking, 53.7% (115) were Certified Smoking Cessation Service Providers (CSCSP).

### Factors associated with types of pharmacy services provided

A total of eleven potential factors which might be associated with the types of pharmacy services provided were analysed using binary logistic regression: sex, age group, ethnic group, education level, local or overseas graduate, CSCSP, MyHealth MyWeight, Harm Reduction Programme, Diabetes Medicine Therapy Adherence Clinic (DMTAC) certified or trained, year of practice and type of employment. Only factors that were significantly associated with the types of pharmacy services provided are shown in Table 2.

**Table 2:** Factors associated with the types of pharmacy services provided (*N* = 409)^a^

Services	Factors	Characteristics	Frequency (%)	Odds Ratio (95% CI)	Adjusted *p*-value	Hosmer-Lemeshow Good of Fit test	
						Chi-square	*p*-value
Home medication review	Education	Basic degree	23 (5.9)	As reference	0.032*	1.986	0.370
		Master	4 (21.1)	3.728 (1.117, 12.447)			
Multiple medicine management	CSCSP	Yes	41 (28.5)	1.848 (1.137, 3.005)	0.013*	0.870	0.929
		No	50 (18.9)	As reference			
Health supplement	Education	Basic degree	357 (91.5)	4.887 (1.699, 14.054)	0.003**	0.742	0.690
		Master	13 (68.4)	As reference			
Pregnancy test	Employment	Self-employed	71 (39.4)	2.803 (1.681, 4.672)	< 0.001**	8.125	0.322
		Full-time employee	33 (14.4)	As reference			
Smoking cessation	Sex	Female	145 (56.2)	2.082 (1.278, 3.392)	0.003**	3.582	0.893
		Male	65 (43.0)	As reference			
	Ethnicity^b^	Malay	34 (40.5)	As reference	0.022*		
		Chinese	167 (54.0)	1.936 (1.100, 3.407)			
	CSCSP	Yes	113 (78.5)	8.158 (4.731, 14.066)	< 0.001**		
		No	97 (36.6)	As reference			
	Employment	Self-employed	73 (40.6)	As reference	0.049*		
		Full-time employee	137 (59.8)	1.642 (1.003, 2.687)			
Weight management	Age	Below 40	127 (56.2)	1.772 (1.118, 2.809)	0.015*	12.542	0.084
		40 and above	89 (48.6)	As reference			
	MyHealth MyWeight	Yes	60 (80.0)	4.892 (2.557, 9.360)	< 0.001**		
		No	156 (46.7)	As reference			

^a^ Eleven respondents from other ethnic groups were excluded from analysis due to insufficient number for statistical comparison

^b^ The three major ethnic groups in Malaysia are Malay, Chinese and Indians but Indians are not shown in this table as there was no significant association when compared to Malay and Chinese

* *p* < 0.05; ** *p* < 0.01

*C*I Confidence interval, *CSCSP* Certified smoking cessation service provider

### Provision of pharmaceutical care services

Of the 420 respondents, 53.3% (224) reported that they were providing pharmaceutical care services to their customers with chronic diseases such as diabetes mellitus (91.5%), hypertension (90.6%), hyperlipidaemia (83.0%), asthma (72.3%) and others (10.7%) which included skin problems, hyperuricaemia and gastrointestinal problems. Most of these respondents reported that they provided pharmaceutical care services to less than 20 patients in the recent one month.

### Factors associated with the provision of pharmaceutical care

Similar factors were analysed using binary logistic regression mentioned. Only demographic factors that were significantly associated with the provision of pharmaceutical care services are shown in Table 3.

**Table 3:** Factors associated with the provision of the pharmaceutical care services (*N* = 409)^a^

Provision	Factors	Characteristics	Frequency (%)	Odds Ratio (95% CI)	Adjusted *p*-value	Hosmer-Lemeshow Goodness of Fit Test	
						Chi-square	*p*-value
Pharmaceutical care service	Ethnic	Malay	32 (38.1)	As reference	-	3.104	0.684
		Chinese	176 (57.0)	2.210 (1.320, 3.698)	0.003**		
		Indian	11 (68.8)	3.988 (1.248, 12.742)	0.020*		
	CSCSP	Yes	88 (61.1)	1.594 (1.032, 2.464)	0.036*		
		No	131 (49.4)	As reference			
	Methadone HRP	Yes	18 (81.8)	3.751 (1.129, 12.461)	0.031*		
		No	201 (51.9)	As reference			

^a^ Eleven respondents from other ethnic groups were excluded from analysis due to insufficient number for statistical comparison

* *p* < 0.05; ** *p* < 0.01

*CSCSP* Certified smoking cessation service provider

*HRP* Harm reduction programme

Table 4 summarises the practice of all the five principles of pharmaceutical care by the respondents. Among the 420 respondents, only 0.95% (4) provided all the components in all the five practice principles of pharmaceutical care by APhA.

**Table 4:** Summary of respondents who fulfilled the practice principles of pharmaceutical care (*N* = 420)

Components of each pharmaceutical practice principle	Frequency (%)	Frequency (%) of practice principle fulfilled
**Patient's Data Collection**		98 (23.3)
Interview patient or caregiver to gather health and medical history	360 (85.7)	
Able to access the patient's medical record from other healthcare providers easily	125 (29.8)	
**Medical Information Evaluation**		78 (18.6)
Ensure that the patient understands his/her current health status	363 (86.4)	
Evaluate the safety and effectiveness of the medicines the patient is using	371 (88.3)	
Documentation of pharmaceutical care issues identified	84 (20.0)	
**Formulating Drug Therapy Plan**		39 (9.3)
Seek to identify, minimise and prevent potential medicine related problems	320 (76.2)	
Recommend to change the patient's medicine regimen when necessary	101 (24.0)	
Advise the patient on choices of medicines within the patient's budget	275 (65.5)	
Documentation of changes of patient's medicine regimen	52 (12.4)	
**Implementing Drug Therapy Plan**		19 (4.5)
Ensure that the patient understands the purpose of the medicines used	410 (97.6)	
Ensure that the patient's medicines are always available in time for patient to use	301 (71.7)	
Contact the patient's other healthcare providers whenever appropriate	42 (10.0)	
Documentation of other interventions	21 (5.0)	
**Monitoring and Modifying Plan and Documentation**		77 (18.3)
Monitor the patient's condition with regular follow-up	184 (43.8)	
Documentation of monitoring plan details	77 (18.3)	

Table 5 shows the factors associated with the practice of components in the five practice principles of pharmaceutical care.

**Table 5:** Factors associated with the components in the five practice principles of pharmaceutical care (*N* = 409)^a^

Practices	Factors	Characteristics	Frequency (%)	Odds Ratio (95% CI)	*p*-value	Hosmer-Lemeshow Goodness of Fit test	
						Chi-square	*p*-value
Interview patient or caretaker	DMTAC	Yes	18 (75.0)	As reference	0.044*	3.781	0.706
		No	334 (86.8)	3.192 (1.033, 9.863)			
Can access information from patient's other health care providers	Ethnic	Malay	17 (20.2)	As reference	0.026*	4.405	0.732
		Chinese	102 (33.0)	1.958 (1.085, 3.531)			
Seek to identify, minimise and prevent medical related problems	Ethnic	Malay	54 (64.3)	As reference	0.002**	1.735	0.884
		Chinese	250 (80.9)	2.370 (1.371, 4.096)			
Recommend changes of medicine when necessary	Ethnic	Malay	10 (11.9)	As reference	-	4.568	0.803
		Chinese	83 (26.9)	2.721 (1.292, 5.732)	0.008**		
		Indian	5 (31.3)	4.253 (1.176, 15.389)	0.027*		
	Employment	Self-employed	58 (32.2)	2.138 (1.312, 3.483)	0.002**		
		Full-time employee	40 (17.5)	As reference			
Advise patient on medicines budget	Ethnic	Malay	44 (52.4)	As reference	0.004**	5.999	0.306
		Chinese	217 (70.2)	2.109 (1.274, 3.491)			
Monitor patient's condition with regular follow up	Age	Below 40	112 (49.6)	1.670 (1.097, 2.542)	0.017*	3.759	0.807
		40 and above	67 (36.6)	As reference			
	Ethnic	Malay	27 (32.1)	As reference	0.032*		
		Chinese	145 (46.9)	1.774 (1.050, 2.995)			
Contact patient's other health care providers	Employment	Self-employed	25 (13.9)	2.305 (1.152, 4.613)	0.018*	2.234	0.897
		Full-time employee	15 (6.6)	As reference			

^a^ Eleven respondents from other ethnic groups were excluded from analysis due to insufficient number for statistical comparison

* *p* < 0.05; ** *p* < 0.01

*DMTAC* Diabetes medicine therapy adherence clinic

### Barriers and facilitators in providing pharmaceutical care services

Table 6 shows the perceived barriers by the respondents in implementing pharmaceutical care services at their community pharmacies.

**Table 6:** Barriers in providing pharmaceutical care services

Areas and statements of barrier	Mean Score (SD)	Number of respondents (%) [*N* = 420]		
		Strongly Disagree and Disagree	Slightly Disagree and Slightly Agree	Agree and Strongly Agree
**Time**				
Lack of time from your side.	4.15 (1.32)	67 (15.9)	146 (34.8)	207 (49.3)
Patient or customer has no time.	4.50 (1.11)	38 (9.0)	115 (27.4)	267 (63.6)
**Space or privacy**				
Lack of space in the pharmacy premises for the patient's privacy.	3.48 (1.46)	143 (34.0)	149 (35.5)	128 (30.5)
**Finances**				
Lack of financial incentive.	3.98 (1.46)	99 (23.6)	127 (30.2)	194 (46.2)
Increased operating cost.	3.91 (1.41)	98 (23.3)	147 (35.0)	175 (41.7)
**Resources**				
Shortage of manpower to provide the service	4.55 (1.16)	34 (8.1)	121 (28.8)	265 (63.1)
Lack of training to provide the service	4.04 (1.35)	78 (18.6)	145 (34.5)	197 (46.9)
Lack of material resources such as computer, internet access, pharmacy software	3.48 (1.44)	138 (32.9)	159 (37.9)	123 (29.2)
Lack of hard copy references such as BNF, MIMS etc.	2.81 (1.29)	222 (52.9)	140 (33.3)	58 (13.8)
**Public awareness**				
Lack of public awareness that such service is available at your community pharmacy	4.40 (1.19)	46 (11.0)	144 (34.3)	230 (54.7)
**Supports from other health care providers**				
Restriction by pharmaceutical suppliers on access of certain medicines to your pharmacy	4.19 (1.26)	55 (13.1)	163 (38.8)	202 (48.1)
Medicine price discrimination by pharmaceutical suppliers.	4.99 (1.09)	21 (5.0)	81 (19.3)	318 (75.7)
Lack of acceptance by doctors on recommendation of medicine regimens by the pharmacist	4.72 (1.05)	17 (4.0)	136 (32.4)	267 (63.6)
**Government or professional health care Policy**				
Lack of dispensing separation which reduces the opportunity for patients who need pharmaceutical care to visit your pharmacy	5.20 (1.01)	14 (3.4)	53 (12.6)	353 (84.0)
Lack of government's initiative for patients to obtain their prescribed medicines from your pharmacy	5.16 (0.99)	13 (3.1)	55 (13.1)	352 (83.8)
Strict enforcement which prevents the supply of follow-up prescription medicines without a valid or complete prescription from doctors	5.00 (1.15)	20 (4.8)	83 (19.8)	317 (75.4)
No standard guidelines by the government or professional bodies on pharmaceutical care services	4.80 (1.11)	23 (5.5)	99 (23.5)	298 (71.0)
**Support from employer (*N* = 233)**^**a**^				
Lack of support from your employer	3.10 (1.40)	100 (42.9)	84 (36.1)	49 (21.0)

^a^Among 420 respondents, only 233 were employed pharmacists

*BNF* British National Formulary, *MIMS* Monthly Index of Medical Specialities

Of the 18 statements on barriers, the lack of separation between prescribing and dispensing had the highest mean score (SD) of 5.20 (1.01) as well as the highest percentage of ‘agree and strongly agree’ response (84.0%). This indicates that community pharmacists perceived this as the greatest barrier for providing pharmaceutical care.

**Fig. 2.**
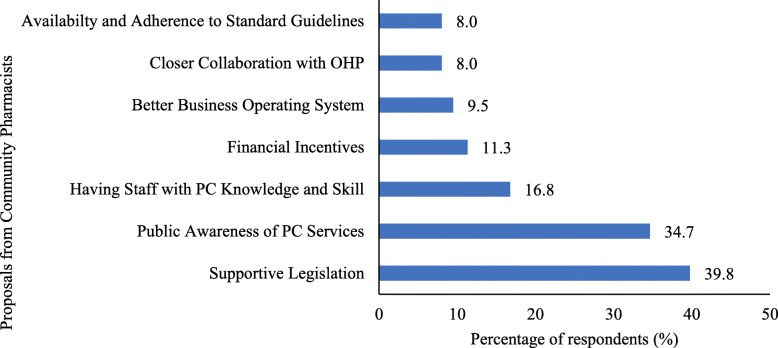
Facilitators for the provision of pharmaceutical care suggested by the community pharmacists (*N* = 274). Note: The percentage may exceed 100% as the respondents can provide more than one suggestion. OHP - Other health care providers; PC - Pharmaceutical care

Of the 420 respondents, 274 (65.2%) suggested measures which may facilitate or encourage the provision of pharmaceutical care at their pharmacies (Fig. 2).

The most common facilitator suggested was for the government to implement supportive legislations which included the separation of prescribing and dispensing, mandatory prescriptions by medical doctors, standardization of medicine prices and zoning of community pharmacies. This was followed by increasing public awareness of pharmaceutical care services provided by community pharmacists.

## Discussion

The present study found that almost all the respondents (99.5%) were providing some types of health screening and monitoring, mainly the measurement of blood pressure, blood glucose and cholesterol levels. This is higher than from a previous nationwide study (77.1%) by Chua et al [16]. This may be attributed to an increase in awareness and demand by the general public [31] especially by those who could not afford home use medical devices [32]. Nevertheless, future studies should examine the effectiveness of such services in detecting and monitoring the prevalence of non-communicable diseases in the country. This will be in line with the Ministry of Health’s consideration of a public-private partnership in Malaysia [24] which will include the transition of care programme where patients with non-communicable diseases can be referred to community pharmacies to reduce the overload in the public health care system.

Most of the respondents (90.5%) also advised their customers on the selection and use of health supplements for general health. This is similar with a study by Chua et al. [13] which reported that the general public in Malaysia approached community pharmacists mainly to purchase health supplements. However, only 15.8% of the respondents who provided such services were accredited with CMEd. In addition, the odds of providing such service among the community pharmacists with a basic pharmacy degree is almost 5.0 times more than those with a master’s degree. Further studies are required to assess the need for such qualifications for more effective counselling on health supplements. Pharmacy programmes in Malaysia should also include topics on health supplements in their curriculum.

More than two-thirds of the respondents were conducting patient medications reviews in their pharmacies and this was 10 times higher than those who were conducting home medication reviews. This may be due to a shortage of pharmacists or staff which is also mentioned by 63.1% of the respondents in the present study and hence, community pharmacists were not able to leave their pharmacies to conduct home medication reviews. Another reason could be a lack of clinical knowledge and skills in providing home medication review as the odds of providing such service among community pharmacists with a master’s degree were 3.7 times more than those with a basic degree in the present study. Further studies on the processes of medication review in the pharmacies or in the homes are required to determine if these were carried out comprehensively as in Australia [33] or other countries [8, 34] which have incorporated the practice principles for pharmaceutical care in their medication review.

About half of the respondents (51.0%) were providing counselling on smoking cessation. This is similar to the findings by Chua et al. (49.1%) [16]. The war against smoking will continue and the demand for advice to quit smoking may increase in future. In addition, only 53.7% of those who were providing counselling on smoking cessation in the present study had undergone the CSCSP programme while the odds of providing this service among those CSCSP was 8.2 times more than those without such certification. Further studies should also be conducted to determine the effectiveness of such service provided by community pharmacists.

Waste management in community pharmacies involved the collection of expired, damaged, or unused medicines and used needles from customers, for proper disposal. A pilot project was initiated in 2018 by the Malaysian Community Pharmacy Guild to enable community pharmacists to educate and assist the general public on the proper disposal of used needles. This was followed by the Green Pharmacy campaign [35] but the present study found that only 7.9% of community pharmacies were providing waste management service. This is as expected since such service is still relatively new and may need some incentives for the community pharmacies as well as more public awareness campaigns.

Multiple medicine management involves the use of medicine dose administration aid that can be provided by community pharmacists to improve medication adherence. This is beneficial especially for elderly patients who are most susceptible to polypharmacy [36]. With the growing ageing population in Malaysia [37], community pharmacists can play a major role in multiple medicines management but the present study showed that not many respondents (22.1%) were offering such service.

Slightly more than half of the respondents (53.3%) reported that they were providing pharmaceutical care services in their community pharmacies, mainly for patients with diabetes, hypertension, hyperlipidaemia and asthma. However, whether these community pharmacists were familiar with the concept and were practising appropriate pharmaceutical care processes were not captured in this study. A study in Brazil reported that community pharmacists who were providing pharmaceutical care did not have adequate knowledge [38].

The present study found that more than two-thirds of the respondents were not able to access patients' medical information from the patients’ medical doctors. Besides a lack of co-operation from the patients' medical doctors, patients also did not allow community pharmacists to access their medical conditions. These findings are similar to that of studies in other developing countries where the general public has limited knowledge on the roles of community pharmacists [39, 40].

The study also revealed that some of the respondents (14.3%) did not gather medical information from their patients or their caregivers before providing their recommendations. Community pharmacists should be more vigilant in responding to patients’ request for treatment as insufficient patients' medical information may lead to inappropriate treatment recommendation and subsequently, may lead to more harm than good to the patients.

Even though more than 80% of the respondents claimed that they evaluated the safety and effectiveness of the medicines the patients were taking and ensured that their patients understood their current health conditions, most did not document such evaluation and its outcomes. Community pharmacists should be reminded on the importance of documentation which is essential for the effective practice of pharmaceutical care.

Most of the respondents (76.2%) reported that they identified, minimised and prevented potential medicine-related problems while formulating a drug therapy plan. Studies in China (93.1%) and Nigeria (91.1%) also showed that majority of the pharmacists identified medicine-related problems when devising pharmaceutical care plans [40, 41].

Only about 25% of the respondents would recommend changes to the patient's medication regimen when required, compared to 55% of community pharmacists in Nigeria [41]. This may be because community pharmacists in Malaysia do not receive many prescriptions with multiple medications since such prescriptions are usually filled in the hospitals or the clinics of general practitioners. Therefore, community pharmacists are not actively involved in the management of patients' medication regimens and in intervening medication-related problems. Similar situations were also observed in Indonesia [42].

Subsequently, only 10% of the respondents would contact their patients’ medical doctors to discuss the need to change the patients’ medication regimens when appropriate and only half of these respondents documented their interventions. These findings are similar with a study in India where majority of the community pharmacists did not contact the patients’ medical doctors on prescription issues [43]. A previous study in Malaysia also found that almost all (98.9%) the medical doctors in private clinics never had any interactions with community pharmacists [44]. These may be attributed to a poor relationship between the two health care professionals [40], and a lack of awareness or acceptance of pharmacists' roles by the prescribers [44–46].

Proper monitoring could be the most important component of pharmaceutical care to detect any undesirable outcomes, but this is usually the least practised [1]. The present study found that less than 20% of the respondents were monitoring and modifying the drug therapy plans for their patients. This finding is similar with studies in Jordan [45] and Saudi Arabia [47].

Community pharmacists in Malaysia rarely document their pharmaceutical care services including the outcomes of their interventions. Poor documentation was also observed in Jordan [45] and Brazil [38]. This may be due to the absence of regulations to keep patients' medical records and also most community pharmacists thought that it was not necessary or were too busy to maintain any documents [48]. Documentation of pharmaceutical services provided should be done for the proper practice of monitoring and modifying of a drug therapy plan.

Ethnicity, employment status and age seemed to determine whether the community pharmacists would conduct the various steps in the practice of pharmaceutical care. Self-employed community pharmacists were more proactive in contacting the patient's other health care providers and recommending changes to the medications. This is as expected since most self-employed individuals will be more enthusiastic and will spend more time with their customers [19]. More in-depth studies such as qualitative studies will be useful to determine how ethnicity affects the provision of pharmaceutical care services.

The present study found that community pharmacists in Malaysia were not providing adequate pharmaceutical care services in the following areas: collaborating with patients' other health care providers to obtain their medical and medication information; managing the patient's medication regimen including the recommendation to change medication when required; having proper patient monitoring and follow-up mechanism; and maintaining documentation of pharmaceutical care services.

The most common barriers that prevented community pharmacists from providing pharmaceutical care services in the present study were government or professional health care policies such as the absence of separation between prescribing and dispensing; government initiative for patients to obtain their prescribed medications from community pharmacies; strict enforcement on the dispensing of prescription-only-medicines with valid prescriptions and lack of standard guidelines on pharmaceutical care services. Lack of supportive health care policies had also been cited as one of the main barriers in other studies [40, 45] including in Malaysia [14] and even in South Korea which practices separation between prescribing and dispensing [29]. The small number of prescriptions received by the community pharmacies in the present study did not provide much opportunity for the provision of pharmaceutical care services to patients with chronic and multiple medications. As suggested by the respondents, the main facilitator for the provision of pharmaceutical care services was having more supportive legislations. The implementation of separation between prescribing and dispensing is a major health care reform which may require political will, time and resources. Perhaps, the authorities concerned can emulate the community pharmacy practice in Thailand where incentives are given to community pharmacists to provide certain services [49].

Another barrier reported was medicine price discrimination by pharmaceutical suppliers. Some medications were not available or were sold to community pharmacies at higher prices [50, 51] and hence, costs of prescribed medications may be higher compared to private hospitals or clinics. This may prevent patients from filling their prescriptions in community pharmacies and consequently less opportunity for prescription screening and for identifying any medication-related problems as well as to provide pharmaceutical care services.

Lack of acceptance by medical doctors for community pharmacists to provide pharmaceutical care services was commonly cited in many developing countries [41, 42, 46]. The present study also found that many respondents (63.6%) reported a lack of acceptance by doctors on recommendation of medication regimens by the pharmacist. This could be due to a lack of doctors’ understanding regarding the clinical knowledge of pharmacists since doctors who had worked closely with community pharmacists trusted these health care professionals [46]. This indicates the importance of open discussion to foster closer collaboration between both health care professionals in providing optimal care to the patients.

### Limitations of the study

The first limitation of this study was that the contact list of community pharmacists may not be complete. Therefore, some community pharmacists might have been missed out and did not have the opportunity to participate in the online self-administered questionnaire. In addition, the study was not able to determine if there is any difference in the characteristics of respondents and non-respondents except for the similar distribution among the various states in Malaysia.

Secondly, as this is the first study on pharmaceutical care services provided by community pharmacists in Malaysia based on the five practice principles for pharmaceutical care stipulated by the APhA [2], the components used to verify each principle may not be foolproof. Nevertheless, this did not affect the findings of the present study where the practice of all these principles by community pharmacists in Malaysia were sub-optimal. More importantly, this study showed the areas of pharmaceutical care services that need improvement by community pharmacists. Future studies should also assess the extent or depth of pharmacy programme curricula in Malaysia which cover such services so that appropriate interventions can be planned and implemented.

## Conclusions

Most community pharmacies in Malaysia were involved in recommending health supplements, conducting patient medication reviews, weight management and smoking cessation services besides common functions such as treating minor ailments, medication dispensing and counselling. The present study found that community pharmacists in Malaysia were not providing adequate pharmaceutical care services compared to international practice principles. Areas that need improvement included collaboration with patients' other health care providers; more proactive management of patient's medicine regimen; having proper patient monitoring and follow-up mechanisms, and documentation. Programmes for the continuous professional development of community pharmacists should include comprehensive training on the provision of pharmaceutical care. Future studies to assess the effectiveness of services provided by community pharmacists especially on patient medication review and pharmaceutical care should be considered. The current government or professional health care policies were perceived by community pharmacists as the main barrier in providing pharmaceutical care services. Authorities concerned should take this into consideration when planning the health care system of the country.

## Supplementary Information

**Additional file 1.**

**Additional file 2.**

## Data Availability

The datasets used and/or analysed for the present study are available from the corresponding author upon reasonable request.

## Abbreviations

APhA: American Pharmacists Association
MPS: Malaysian Pharmaceutical Society
SD: Standard deviation
IQR: Interquartile range
CMEd: Complementary medicine education
CSCSP: Certified smoking cessation service provider
HRP: Harm reduction programme
DMTAC: Diabetes medicine therapy adherence clinic
CI: Confidence interval
BNF: British national formulary
MIMS: Monthly index of medical specialities
OHP: Other health care providers
PC: Pharmaceutical care
n.d.: no date
