# Supplementary material for: The extent and barriers in providing pharmaceutical care services by community pharmacists in Malaysia: a cross-sectional study
Source: BMC Health Serv Res. 2021 Aug 16;21:822. doi: 10.1186/s12913-021-06820-7 (PMC8365940; doi:10.1186/s12913-021-06820-7)
Supplement: Supplementary file 1 — Additional file 1. [file 12913_2021_6820_MOESM1_ESM.pdf]

Serial No. ________________

Date: ________________

Title:

Pharmaceutical Care Services Provided by Community Pharmacists in Malaysia

Section A: Demographic Data

1. Are you working as a full-time community pharmacist in the private sector, NOT in the administration department or community pharmacy in the hospital premises?

☐ Yes (Please proceed to answer the following questions).

☐ No (This survey is not for you and you do not have to participate in this survey, thank you.)

2. Sex:

☐ Female

☐ Male

3. Year of birth: __________________

4. Ethnic Group:

☐ Malay

☐ Chinese

☐ Indian

☐ Others (please specify): ____________________________

5. Highest education level in Pharmacy related areas:

☐ Basic Pharmacy Degree

☐ Postgraduate Master in Clinical Pharmacy or Pharmacy Practice

☐ Doctor of Philosophy in Clinical Pharmacy or Pharmacy Practice

6. Which country did you obtain your Basic Pharmacy Degree?

Answer: __________________________

7. Do you have any other postgraduate certificates or postgraduate diplomas?

☐ None

Yes (you can select more than one answer)

☐ Certified Smoking Cessation Service Provider (CSCSP)

☐ Weight Management - MyWeight MyHealt Program Service Provider

☐ Harm Reduction Program - Methadone Dispensing

☐ Medication Therapy Adherence Clinic (MTAC) Service Provider - Diabetes

☐ Others (please specify:)

8. Do you own or partially own the Community Pharmacy that you are working in?

☐ Yes

☐ No

9. How many years have you been working as a Community Pharmacist in Malaysia?

Answer: _______________

10. Location of your Community Pharmacy:

☐ Johor

☐ Kedah

☐ Kelantan

☐ Labuan

☐ Melaka

☐ Negeri Sembilan

☐ Pahang

☐ Penang

☐ Perak

☐ Perlis

☐ Putrajaya

☐ Sabah

☐ Sarawak

☐ Selangor

☐ Wilayah Persekutuan

11. Please state the town/city where your Community Pharmacy is located (eg. Seremban, Muar, etc):

Answer: ________________________________

Section B: Customers Services By Community Pharmacists

12. Besides treating minor illnesses, dispensing medicines and providing counselling for medication use, which of the following services are conducted by YOU OR YOUR STAFF on a REGULAR BASIS to your patients or customers ?

Please tick the appropiate box:	Yes	No
i. Patient Medication Review<br>Conduct Medication Review at your pharmacy.	☐	☐
ii. Home Medication Review<br>Conduct Home Medication Review outside your pharmacy.	☐	☐
iii. Multiple Medicines Management<br>Multi-dose Medicine Packaging System or similar service for patients who need multiple medicines management.	☐	☐
iv. Dietary Health Supplements<br>Health supplement selection and recommendation for general health.	☐	☐
v. Pregnancy Test<br>Conduct pregnancy test at your pharmacy.	☐	☐
vi. Alternative Treatment<br>Provide alternative treatment method, such as acupunture and traditional or complementary medicines i(e. Blackmores CMed).<br>Please specify these alternative methods: ______________________	☐	☐
vii. Extemporaneous Preparation<br>Prepare extemporaneous preparation or compound medicines for specific patients.	☐	☐
viii. Methadone Dispensing<br>Provide methadone replacement therapy under Ministry of Health Malaysia.	☐	☐
ix. Smoking Cessation Programme<br>Counsel or help customers to quit smoking.	☐	☐

x. Weight Management
Provide weight management advice and weight control programme. ☐ ☐

xi. Waste Management
Collect expired, damaged or unused medicines from customers, for proper disposal. ☐ ☐

xii. Others (please specify:) ________________________________

13. Which of the following Screening and Monitoring Services are conducted by YOU OR YOUR STAFF at your community pharmacy on a REGULAR BASIS ?

Please tick the appropiate box:	Yes	No
i. Blood Pressure	☐	☐
ii. Blood Glucose	☐	☐
iii. HbA1c	☐	☐
iv. Blood Cholesterol	☐	☐
v. Blood Uric Acid	☐	☐

vi. Others (please specify:) __________________________

Section C : Pharmaceutical Care Services

14. Do you provide Pharmaceutical Care services to your patients with chronic diseases?

Please tick the appropiate box: Yes ☐ No ☐

i. If Yes for ALL patients with chronic diseases, please state the chronic disease(s) and the number of cases in the recent one month (i.e. Diabetes - 3):

____

____

ii. If Yes for SELECTED patients with chronic diseases, please state the chronic disease(s) and the number of cases in the recent one month (i.e. Diabetes - 3):

____

____

15. Which of the following services are you providing to those patients with chronic diseases?

	Please tick the appropiate box:	Yes	No
i.	Interview patient or caregiver to gather his/her health and medical history.	☐	☐
ii.	When needed, with patient's consent, you can access the patient's medical record from his/her other healthcare providers easily.	☐	☐

If No, please state why: ____

		Yes	No
iii.	Evaluate the safety and effectiveness of the medicines the patient is using.	☐	☐
iv.	Seek to identify, minimize and prevent potential medicine-related problems.	☐	☐
v.	Change the patient's medicine regimen when necessary.	☐	☐

If yes, please state the NUMBER of cases in the RECENT one month: ______________________

		Yes	No
vi.	Contact the patient's other healthcare providers (eg. his/her doctors) to discuss the need to change the patient's medicine regimen whenever appropiate.	☐	☐

If yes, please state the NUMBER of cases in the RECENT one month: ______________________

vii.	Ensure that the patient understands the purpose of the medicines used.	☐	☐
viii.	Ensure that the patient understands his/her current health status.	☐	☐
ix.	Ensure that the patient's medicines are always available in time for him/her to use.	☐	☐
x.	Advise patient on choices of medicines within patient's budget.	☐	☐
xi.	Monitor the patient's condition with regular (eg. one a month) follow-up.	☐	☐

16. Do you RECORD the following action taken?

Please tick the appropiate box:	Yes	No
i. Pharmaceutical Care issues identified.	☐	☐
ii. Changes of patient's medicine regimen.	☐	☐
iii. Other interventions (i.e. contacted the prescribers or doctor concerned).	☐	☐
iv. Monitoring plan details.	☐	☐

17. Please state the NUMBER of prescriptions you received from doctors for the PAST ONE YEAR ?

Answer: _______________________

Section D: Barriers in Providing Pharmaceutical Care at Community Pharmacy

For each statement below, please click the answer on a scale of 0 (Strongly Disagree) to 5 (Strongly Agree) that best represents what you think as the BARRIERS in providing Pharmaceutical Care services in YOUR community pharmacy:

Statement	0	1	2	3	4	5
**18. Time**						
i. Lack of time from your side.	Strongly Disagree	Disagree	Slightly Disagree	Slightly Agree	Agree	Strongly Agree
ii. Patient or customer has no time.	Strongly Disagree	Disagree	Slightly Disagree	Slightly Agree	Agree	Strongly Agree
**19. Space or Privacy**						
i. Lack of space in the pharmacy premise for patient's privacy.	Strongly Disagree	Disagree	Slightly Disagree	Slightly Agree	Agree	Strongly Agree
**20. Finance or Commercial Objectives**						
i. Lack of financial incentive.	Strongly Disagree	Disagree	Slightly Disagree	Slightly Agree	Agree	Strongly Agree
ii. Increased operating cost.	Strongly Disagree	Disagree	Slightly Disagree	Slightly Agree	Agree	Strongly Agree
**21. Resources**						
i. Shortage of manpower to provide the service.	Strongly Disagree	Disagree	Slightly Disagree	Slightly Agree	Agree	Strongly Agree
ii. Lack of training to provide the service.	Strongly Disagree	Disagree	Slightly Disagree	Slightly Agree	Agree	Strongly Agree
iii. Lack of material resources such as computer, internet access, pharmacy software, ...etc	Strongly Disagree	Disagree	Slightly Disagree	Slightly Agree	Agree	Strongly Agree
iv. Lack of hard copy references such as BNF, MIMS...etc	Strongly Disagree	Disagree	Slightly Disagree	Slightly Agree	Agree	Strongly Agree

22. Marketing

i. Lack of public awareness that such service is available at your community pharmacy.	Strongly Disagree	Disagree	Slightly Disagree	Slightly Agree	Agree	Strongly Agree

23. Support from Other Healthcare Providers

i. Restriction by pharmaceutical suppliers on access of certain medicines to your pharmacy.	Strongly Disagree	Disagree	Slightly Disagree	Slightly Agree	Agree	Strongly Agree
ii. Medicine price discrimination by pharmaceutical suppliers.	Strongly Disagree	Disagree	Slightly Disagree	Slightly Agree	Agree	Strongly Agree
iii. Lack of acceptance by doctors on recommendation of the medicine regimens by pharmacist.	Strongly Disagree	Disagree	Slightly Disagree	Slightly Agree	Agree	Strongly Agree

24. Government or Professional Healthcare Policy

i. Lack of dispensing separation which reduces the opportunity for patients who need pharmaceutical care to visit your pharmacy.	Strongly Disagree	Disagree	Slightly Disagree	Slightly Agree	Agree	Strongly Agree
ii. Lack of government's initiative for patients to obtain their prescribed medicines from your pharmacy.	Strongly Disagree	Disagree	Slightly Disagree	Slightly Agree	Agree	Strongly Agree
iii. Strict enforcement which prevents the supply of follow-up prescription medicines without a valid or complete prescription from doctors.	Strongly Disagree	Disagree	Slightly Disagree	Slightly Agree	Agree	Strongly Agree
iv. No standard guidelines by the government or professional bodies on pharmaceutical care services.	Strongly Disagree	Disagree	Slightly Disagree	Slightly Agree	Agree	Strongly Agree

25. Other barriers (please specify:) ____

26. Ownership (question FOR EMPLOYED PHARMACISTS ONLY)

i. Lack of support from your employer.	Strongly Disagree	Disagree	Slightly Disagree	Slightly Agree	Agree	Strongly Agree

27. What can be done to promote or encourage the provision of Pharmaceutical Care services at your pharmacy (if any)?

____

____

____

____

____

____
