# Supplementary material for: The extent and barriers in providing pharmaceutical care services by community pharmacists in Malaysia: a cross-sectional study
Source: BMC Health Serv Res. 2021 Aug 16;21:822. doi: 10.1186/s12913-021-06820-7 (PMC8365940; doi:10.1186/s12913-021-06820-7)
Supplement: Supplementary file 2 — Additional file 2. [file 12913_2021_6820_MOESM2_ESM.docx]

**Supplementary Table: Fulfilment of pharmaceutical care practice principles**

| **Pharmaceutical Care Practice Principle** | **Sub-questions in the questionnaire to access the practices of pharmaceutical care** | **Positive Response Required** |
| --- | --- | --- |
| 1. Patients' Data Collection | (1) Interview patient or caregiver to gather his/her health and medical history. | All |
|  | (2) When needed, with the patient's consent, you can access the patient's medical record from his/her other health care providers easily. |  |
| 2. Medical Information Evaluation | (3) Ensure that the patient understands his/her current health status. | All |
|  | (4) Evaluate the safety and effectiveness of the medicines the patient is using. |  |
|  | (5) Do you RECORD or DOCUMENT the following action taken: (Pharmaceutical Care issues identified)? |  |
| 3. Formulating A Drug Therapy Plan | (6) Seek to identify, minimise and prevent potential medicine-related problems. | All |
|  | (7) Recommend to change the patient's medicine regimen when necessary. |  |
|  | (8) Advise the patient on choices of medicines within the patient's budget. |  |
|  | (9) Do you RECORD or DOCUMENT the following action taken: (Changes of patient's medicine regimen)? |  |
| 4. Implementing A Drug Therapy Plan | (10) Ensure that the patient understands the purpose of the medicines used. | All |
|  | (11) Ensure that the patient's medicines are always available in time for him/her to use. |  |
|  | (12) Contact the patient's other health care providers (eg. his/her doctors) to discuss the need to change the patient's medicine regimen whenever appropriate. |  |
|  | (13) Do you RECORD or DOCUMENT the following action taken: (Other interventions, i.e. contact the prescriber or doctor concerned)? |  |
| 5. Monitoring and Modifying The Plan | (14) Monitor the patient's condition with regular (eg. one a month) follow-up. | All |
|  | (15) Do you RECORD or DOCUMENT the following action taken: (Monitoring plan details)? |  |
